# Measuring rurality in health services research: a scoping review

**DOI:** 10.1186/s12913-022-08678-9

**Published:** 2022-11-12

**Authors:** Robin Danek, Justin Blackburn, Marion Greene, Olena Mazurenko, Nir Menachemi

**Affiliations:** 1 Indiana University School of Medicine-Terre Haute, 1433 N 6 ½ St., Terre Haute, IN 47802 USA; 2 Richard M. Fairbanks School of Public Health, Indiana University Richard M, Indianapolis, IN, USA; 3 Regenstrief Institute, Indianapolis, IN, USA

**Keywords:** Health services research, Literature review, Rural health, Health policy

## Abstract

**Purpose:**

This study is a scoping review of the different methods used to measure rurality in the health services research (HSR) literature.

**Methods:**

We identified peer-reviewed empirical studies from 2010–2020 from seven leading HSR journals, including the Journal of Rural Health, that used any definition to measure rurality as a part of their analysis. From each study, we identified the geographic unit (e.g., county, zip code) and definition (e.g., Rural Urban Continuum Codes, Rural Urban Commuting Areas) used to classify categories of rurality. We analyzed whether geographic units and definitions used to classify rurality differed by focus area of studies, including costs, quality, and access to care. Lastly, we examined the number of rural categories used by authors to assess rural areas.

**Findings:**

In 103 included studies, five different geographic units and 11 definitions were used to measure rurality. The most common geographic units used to measure rurality were county (*n* = 59, 57%), which was used most frequently in studies examining cost (*n* = 12, 75%) and access (*n* = 33, 57.9%). Rural Urban Commuting Area codes were the most common definition used to measure rurality for studies examining access (*n* = 13, 22.8%) and quality (*n* = 10, 44%). The majority of included studies made rural versus urban comparisons (*n* = 82, 80%) as opposed to focusing on rural populations only (*n* = 21, 20%). Among studies that compared rural and urban populations, most studies used only one category to identify rural locations (*n* = 49 of 82 studies, 60%).

**Conclusion:**

Geographic units and definitions to determine rurality were used inconsistently within and across studies with an HSR focus. This finding may affect how health disparities by rural location are determined and thus how resources and federal funds are allocated. Future research should focus on developing a standardized system to determine under what circumstances researchers should use different geographic units and methods to determine rurality by HSR focus area.

**Supplementary Information:**

The online version contains supplementary material available at 10.1186/s12913-022-08678-9.

## Introduction

According to some federal estimates, nearly 1 in 5 Americans live in rural areas [1]. In general, compared to their urban counterparts, rural Americans are older [2], more likely to be disabled or a veteran of the US military [3, 4], receive Medicaid or be uninsured [5], and have lower median incomes [6]. Rural Americans also have higher rates of obesity [7], cardiovascular diseases [8], and substance use disorders [9]. However, as policymakers become increasingly interested in addressing health disparities between urban and rural populations, it is important to assess and evaluate the different methods used to define rurality so that true disparities can be accurately captured and addressed.

There is no standard way to measure ‘rurality’ or what qualifies as a ‘rural’ area. Even within the US government, multiple definitions of rurality exist which contributes to variability in federal estimates of the size of the rural population. For example, the US Department of Agriculture estimates there are 46 million rural Americans (14%), while the Census Bureau estimates there are nearly 60 million rural residents (18%) resulting in an almost 30% relative difference [10, 11]. Likewise, health services researchers use multiple geographic units and definitions to measure rurality, such as county [12–14], zip code or rural–urban commuting patterns [13–17]. Importantly, some key outcome measures, such as access to care estimates and the incidence of breast cancer were shown to be sensitive to the rural measurement method used [18]. Inconsistent usage of these definitions can influence policy decisions, as demonstrated by Kozhimannil et al., (2018) who documented loss of obstetric services in rural areas. In their study, researchers were unable to use appropriate measurements of rurality because the dataset restricted them to using county only, which masked differences in loss of services within counties that varied in their degrees of rurality [19]. Likewise, other research has indicated significant variability in how rurality is measured across social and health sciences [20]. Over time, several calls have been made to better understand how rurality is measured and to move towards standardization in measures of rurality in health services research (HSR) [12, 15, 20–25]. Although the selection of a rural definition might be a function of data availability, this creates challenges in generalizability and comparability across studies, making policy development difficult [24].

The purpose of the current study is to identify and describe the different definitions used to measure rurality in health services literature and to determine the frequency in which each definition is used. In addition, we stratify studies based on their focus area to determine whether the type of rural definition used is consistent within similar topics across studies. Each definition and measurement approach may have benefits and drawbacks that are not fully understood in the process of policy development. Ultimately, because the definition used to measure rurality can affect how conclusions are drawn, our study will be useful to policymakers, researchers, and other stakeholders interested in addressing health disparities in rural areas.

## Methods

Our approach follows the general guidelines of the Preferred Reporting Items for Systematic Reviews and Meta-Analyses (PRISMA) [25]. Although our study is not technically a systematic review, we apply similar methods to identify, screen and include articles for analysis. Specifically, we included peer-reviewed empirical studies from the HSR literature that used any method to measure rurality as a part of their analysis. Thus, we included studies that analyzed and described differences between rural and urban populations and studies that focused solely on rural populations.

For the current study, we focused on the health services research, including the delivery of health care services to rural populations, which previous research has shown to be problematic in rural areas and different for rural populations than for urban [26, 27]. Our inclusion criteria included empirical publications where the primary dependent variable is consistent with studies that meet the criteria for HSR, as it is defined by the Agency for Healthcare Research and Quality (AHRQ), the leading federal funding agency of studies aimed at improving the performance of health care. According to AHRQ, HSR includes a “multidisciplinary field of scientific investigation that studies how social factors, financing systems, organizational structures and processes, health technologies and personal behaviors affect access to health care, the quality and cost of health care, and ultimately, our health and well-being [28]”. Since the focus was on empirical studies, we excluded letters to the editors, commentaries, case studies, executive summaries and non-peer-reviewed governmental reports.

For our analysis, we identified studies published from 2010 to 2020 that met the inclusion criteria from seven leading HSR journals as identified by prior research, including the Journal of Rural Health [29]. The Journal of Rural Health is a peer-reviewed journal focused on research examining rural health policy, health care delivery, and population health. The other included journals were Health Affairs, Medical Care, Health Services Research, the American Journal of Public Health, Medical Care Research and Review, and the Journal of Healthcare Management. In these journals, we searched for the word “rural” and “rurality” in titles and abstracts to identify studies for further assessment. Because the Journal of Rural Health exclusively publishes studies conducted in rural populations and related topics, we excluded the words “rural” from our search, and instead used HSR terms of “cost,” “quality,” and “access” in our search strategy.

**Fig. 1:**
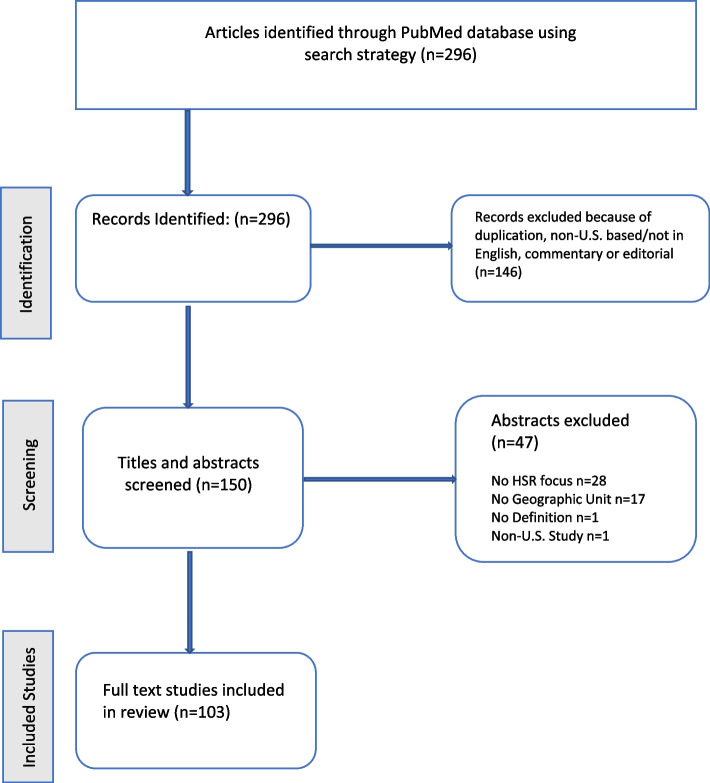
Flow Diagram of Included Studies

Once our initial sample was identified, titles were screened by a single researcher (RD) to determine their eligibility for inclusion in the study (see Fig. 1: Flow Diagram of Included Studies). Studies that were commentaries or editorials, conducted outside of the United States, and did not use an HSR outcome (e.g., quality, cost, access) as a primary focus were eliminated. Journals that cater to international health services research are not likely to use US definitions of rurality and were therefore excluded. An additional author (NM) screened a random subsample of final abstracts and fully agreed on the extracted variables, thus minimizing concerns of intercoder reliability.

For each article that met all the inclusion criteria, we identified the specific geographic unit and definition used to measure rurality. In order to be included in the final sample, each study had to specify at least the geographic unit used to measure rurality (e.g., county, zip code, etc.) or a definition used to determine rurality (e.g., Rural Urban Continuum Codes, Urban Influence Codes). Once the geographic unit and definitions were determined, each study was categorized into 4 broad categories including costs, quality of care, access to care, or ‘other’ topics. Studies coded as ‘other’ included those that focused on the organizational structure of rural hospitals, rural public health delivery systems, the nursing workforce, or workforce issues in Critical Access Hospitals but did not fit into the main HSR categories. Additionally, we extracted the type of data analyzed (primary or secondary), whether the study compared rural and urban populations or focused exclusively on rural populations.

To analyze the data, we calculated the frequency in which each measurement of rurality appears among included studies and within each HSR category. Likewise, we also determined whether study characteristics, as described above, are associated with specific definitions used to measure rurality. We conducted Chi-square analyses to explore how key article characteristics are related to measurements of rurality used by authors. The Institutional Review Board at Indiana University determined this study was exempt from human subject’s oversight. All analyses were performed in Stata version 16.

## Results

Our search strategy resulted in a sample of 296 studies, of which 103 met our inclusion criteria. The majority of included studies made rural versus urban comparisons (*n* = 82, 80%) as opposed to focusing on rural populations only (*n* = 21, 20%) (see Table 1). Among studies that compared rural and urban populations, most studies used only one category to identify rural locations (*n* = 49 of 82 studies, 60%). More than half of the studies were categorized as focusing on access (*n* = 57, 55%), followed by quality (*n* = 23, 22%), and costs (*n* = 16, 16%). Almost all of the included studies (90%) used secondary data sources.

**Table 1:** Description of studies with health services research focus in rural populations included in the analysis (*n* = 103)

Variable	Frequency (%)
**Geographic Unit**	
County	59 (57%)
Zip Code	36 (35%)
Population Density	4 (4%)
Other	4 (4%)
**Definition Used to Determine Rurality**	
Rural–Urban Commuting Area (RUCA) Codes	30 (29%)
Metropolitan Statistical Areas	16 (15%)
Rural–Urban Continuum Codes (RUCC)	12 (12%)
Urban Influence Codes	11 (11%)
County Designation (state or federal)	11 (11%)
Core Based Statistical Areas	6 (6%)
National Center for Health Statistics approach	6 (6%)
Other^a^	11 (11%)
**Population Studied**	
Rural and Urban	83 (81%)
Rural Only	20 (19%)
**Number of Rural Categories used by authors**	
1	66 (64%)
2	20 (19%)
3 or more	17 (17%)
**Data Type**	
Primary	10 (10%)
Secondary	93 (90%)
**Primary HSR Focus**	
Access	57 (55%)
Quality	23 (22%)
Cost	16 (16%)
Other^a^	7 (7%)
**Studies Found Difference in Outcomes Between Urban and Rural Populations (yes)**	68 (92%)
**Year**	
2010 – 2013	17 (17%)
2014—2017	35 (34%)
2018—2020	44 (46%)
**Journal**	
Journal of Rural Health	44 (43%)
Health Affairs	21 (20%)
Medical Care	12 (11%)
Health Services Research	10 (10%)
American Journal of Public Health	9 (9%)
Medical Care Research and Review	5 (5%)
Journal of Healthcare Management	2 (2%)

^a^Other includes the Veterans Administration classification, Federal Office of Rural Health Policy’s classification, geocoding and Zip Code tabulation areas [29]

The most common geographic units used to measure rurality were county (57%) or zip code (35%). The use of population density (4%) and other geographic units of measurement (2%) were uncommon among included studies. With respect to the definition used to determine rurality, the most common approaches utilized were Rural Urban Commuting Area codes (29%) and Metropolitan Statistical Areas (15%). Less common approaches included Rural Urban Continuum Codes (12%), Urban Influence Codes (11%), or the use of a state or federal county designation (11%) to determine rural locations. Overall, 11 different methods to determine rurality were identified among included studies (see Table 1).

We present bivariate crosstabulations between HSR focus area (e.g., cost, quality, access) and geographic unit in Table 2, and between HSR focus area and definition used in Table 3. As shown in Table 2, the geographic unit of county was most commonly used in studies that focused on cost (75%), access (57.9%), and other HSR outcomes (71.4%). The most common geographic unit used among studies focused on quality was zip code (47.8%). In contrast, as seen in Table 3, there was no clear pattern in the use of definition to determine rurality across studies with a different HSR focus.

**Table 2:** Focus of included studies and Geographic Unit used to measure rurality (*n* = 103 articles)

Geographic Unit	Cost	Quality	Access	Other
Zip Code	4 (25.0%)	11 (47.8%)	19 (33.3%)	2 (28.6%)
County	12 (75%)	9 (39.1%)	33 (57.9%)	5 (71.4%)
Population Density	-	1 (4.3%)	3 (5.3%)	-
Other	-	2 (8.7%)	2 (3.5%)	-
Total	16 (100%)	23 (100%)	57 (100%)	7 (100%)

**Table 3:** Focus of included studies and definition used to determine rurality (*n* = 103 articles)

Method used to determine Rurality	Cost	Quality	Access	Other
Rural Urban Continuum Codes	2 (12.5%)	1	8 (14.0%)	1 (14.3%)
Rural–Urban Commuting Area Codes	4 (25%)	10 (44%)	13 (22.8%)	3 (42.9%)
Urban Influence Codes	4 (25%)	3 (13%)	4 (7.0%)	0
County Designation (state and federal level)	2 (12.5%)	3 (13%)	4 (7.0%)	2 (28.5%)
Core Based Statistical Areas	-	1 (4%)	5 (8.8%)	-
Metropolitan Statistical Areas	3 (18.7%)	-	12 (19.3%)	1 (14.3%)
National Center for Health Statistics	-	1 (4%)	5 (8.8%)	-
Other	1 (6.3%)	4 (22%)	6 (12.3%)	-
Total	16 (100%)	23 (100%)	57 (100%)	7 (100%)

Among the 82 studies that compared rural and urban areas, 91% reported a difference in primary outcome by geographic location. In Table 4, we examined whether studies that reported a significant difference between rural and urban areas differed with respect to geographic unit, the definition of rurality, and number of categories used to categorize rural locations. No significant differences were identified.

**Table 4:** Relationships between studies that found a difference in outcomes between urban and rural populations and various key variables (*n* = 82)

Variable	Studies Found Difference in Outcomes Between Urban and Rural Populations	
	Yes	No
**Geographic Unit**		
County	44 (59%)	4 (57%)
Zip Code	26 (30%)	3 (29%)
Population Density	2 (3%)	0
Other	3 (8%)	0 (14%)
**Definition Used to Determine Rurality**		
Rural–Urban Commuting Area (RUCA) Codes	23 (31%)	3 (43%)
Rural–Urban Continuum Codes (RUCC)	10 (12%)	1 (14%)
Urban Influence Codes	7 (9%)	2 (29%)
County Designation (state or federal)	5 (7%)	0
Core Based Statistical Areas	6 (8%)	0
Metropolitan Statistical Areas	10 (12%)	1 (14%)
National Center for Health Statistics approach	5 (7%)	0
Other	9 (14%)	0
**Number of Rural Categories**		
1	42 (56%)	6 (86%)
2	19 (25%)	0
3 or more	14 (16%)	1 (14%)
**Year**		
2010 – 2013	13 (17%)	2 (29%)
2014—2017	25 (33%)	1 (14%)
2018—2020	37 (50%)	4 (57%)

## Discussion

After assessing HSR studies focused on rural health, we found that five different geographic units and eleven definitions were used to measure rurality. Among the geographic units used, county was the most common representing more than half of all studies. Among the definitions used to determine rurality, RUCA was the most common, but was used in less than one-third of studies, highlighting the variability in definitions used by researchers. Prior research has described the different ways that rurality has been measured globally—and how these measurements have evolved over time [20]. Our US-based study, focuses on measurements of rurality by HSR foci and highlights inconsistences in definitions utilized by the HSR focus of included studies. Among studies focused on costs, county was the most common geographic unit, whereas for studies that focused on access, zip codes were the most frequently used geographic unit. Likewise, there were different patterns in the use of methodological approaches by articles with different HSR foci.

Relying heavily on the use of county as the geographic unit to determine rurality can be problematic given that county-level measurement can undercount rural locations [24]. In particular, this criticism is commonly noted of the US Office Management and Budget (OMB) who created Metropolitan and Micropolitan Statistical Areas (MSAs), a commonly used measure of rurality, to classify counties as either urban or rural respectively. Metropolitan areas are defined as an area with a large population nucleus surrounded by adjacent communities, whereas a Micropolitan area is defined as an area with a smaller nucleus [30]. As the OMB cautions, this definition excludes rural areas that reside within large counties that possess urbanized areas elsewhere. In fact, the OMB advises explicitly against using their approach to determine program funding, lest rural programs be overlooked using their classification system to determine rurality in a metro area. This is an important point for researchers to consider, as inappropriate usage of geographic measurements and methods can lead to spurious conclusions about outcomes related to HSR studies of rural areas.

In the current review, we found that RUCA codes were the most commonly used definition to determine rurality, although this definition was used in less than one-third of included studies. Unlike other definitions, RUCA codes are based on census tracts but have been converted to ZIP codes. RUCA codes employ a far more nuanced classification system in which zip codes are used to make up 10 primary codes and 33 secondary codes that in turn are categorized into 6 different classification systems, A through F [30], based on how many different levels of rurality are needed. The majority of included studies used only one measure of rurality which fails to make use of the multiple levels of rurality afforded by the use of RUCA codes. Similarly, Urban Influence Codes divide nonmetro areas into twelve different codes based on adjacency to metro and nonmetro areas in their classification scheme, although this method was used far less frequently than RUCA codes overall—and were frequently used with binary measures of rural location as well. In many cases, either of these two methods may be particularly beneficial to rural HSR researchers, as they provide a currently missed opportunity to examine the effect of HSR outcomes at several different levels of rurality, rather than just by one single category, as commonly observed in our sample.

We found that studies with different HSR foci were inconsistent in their usage of geographic units and definitions to determine rurality. Studies whose HSR focus was access used county most frequently as its geographic unit of measurement, an arguably less precise measure than zip code, as counties may cover a larger expanse of land and obscure difficulties accessing care in larger, urban centers for rural populations. This is an important finding as this may indicate that HSR studies focused on cost or quality may be better suited to use county as their unit of measurement than access studies where adjacency to urban centers that offer more health care services is particularly important. When studies focused on access utilize county as their geographic unit, it is incumbent on the researcher to justify their method because county fails to measure adjacency reliability. The use of zip codes may provide a more granular, and thus a more precise way to measure access to care in rural areas.

In almost all included studies, the authors did not provide an explanation for why they chose a particular method of rurality. Their decision to use a specific approach may be a function of data availability, given that most included studies used secondary data sets. More research is needed to determine how many approaches to measure rurality are possible with commonly used datasets used by authors conducting rural research. Another potential solution proposed by previous research is enabling researchers from institutions outside of federal agencies to access more granular geographic data than commonly available in large, nationally representative datasets (Zhand et al., 2019) [31]. In their study of rural cancer disparities, Zahnd et al. (2019) note that enabling researchers to access more granular geographic data may lead them to use more appropriate definitions of rurality than is currently available in the limited publicly available data sets. Given that authors are rarely given a choice in definitions, they should be encouraged to explicitly state why their chosen methods to measure rurality were used, including if it was the only available option. Moreover, others have suggested that researchers consider the region when appropriate, when determining what method of rurality to use for their analyses [32]. In cases where the dataset used offers multiple options for determining rurality, researchers should justify their primary approach and conduct a sensitivity analysis to state whether their conclusions would change if any alternative approaches are used.

The majority of included studies used only one category to measure rural locations and therefore may be missing important differences in health outcomes when comparing degrees of ruralness and rural and urban populations. Furthermore, we found no consistent or discernable pattern when the number of categories of rurality varied in included studies. Likewise, some authors used unusual descriptors such as ‘highly rural,’ [33, 34] ‘isolated rural’, or super rural, which makes it further challenging to synthesize findings across studies that use different categorizations and terminology as opposed to using currently existing [35–38]. In order to address this issue, researchers should consider weighting data sets in order to create urban and rural populations that are representative of the populations being studied, and thus making it easier to compare different populations in analyses. Additionally, when faced with using multiple categories of rurality with small cell sizes, researchers should consider collapsing rural categories into more than one category, avoiding creating two dichotomous urban–rural categories frequently used in research involving rural populations. Researchers may also consider conducting additional analyses using urban/rural categories and then comparing results to when multiple, more granular categories of rurality are used.

We note that most definitions of rurality are defined in terms of not being metropolitan, as opposed to being defined by a set of criteria completely separate from urbanicity. In fact, when defining rural–urban measurements, the metropolitan groupings are usually defined first, with rural areas making up ‘everything else’ that isn’t considered metropolitan or micropolitan. This is problematic because what is considered rural can vary drastically depending on the definition of “metropolitan,” as evident by vast differences between definitions of rurality. This can lead to problems evaluating rural research even within HSR because inconsistent measurements of rurality can lead to heterogeneity of results due to arbitrary measurements.

Our findings suggest that the tools used to measure rurality are used inconsistently, potentially leading to spurious results and conclusions. This is particularly concerning as the measurements used to determine rurality and what qualifies as rural can affect the allocation of federal funds for rural areas. This is especially important as it relates to access to care and funding to improve health care access or program planning for rural areas [25]. Likewise, findings from this study may encourage local and state policymakers to use more granular definitions of rurality, such as zip code, to design programs that target areas in high need.

There are several limitations to our study worth noting. First, our search strategy may have limited the number of studies included. We recognize that many rural HSR studies exist that are not published in the journals we focused upon, including journals that were not identified as among the top HSR journals or were published in clinical or medical journals. Second, our search yielded only 103 included studies, which made conducting more sophisticated analyses to examine associations between health outcomes and geographic units of measurement and methods to determine rurality not possible. Lastly, we recognize the potential for publication bias to have affected our conclusions. Publication bias occurs when journals favor the publication of studies which report statistically significant results. Thus, it is possible that studies with null findings—especially if it is a function of rural measurement– are underrepresented in the literature and therefore excluded from our analysis.

In conclusion, we found that the geographic units and definitions to determine rurality were used inconsistently within and across studies with an HSR focus area. The use of effective measures of rurality have implications to both rural health policy and additional HSR research that builds upon a presumably known relationship between measurements of rurality and health outcomes in the literature. Tools used to measure rurality can affect how conclusions about health disparities are determined and, in turn, how funds are allocated to programs in rural areas. Future research should focus on developing a standardized system to determine under what circumstances HSR researchers should use different geographic units and methods to determine rurality by HSR focus area.

## Supplementary Information

**Additional file 1. **Appendix of included studies.

## Data Availability

The dataset generated and analyzed during the current study are available at https://www.datafiles.samhsa.gov/dataset/nsduh-2002-2018-ds0001-nsduh-2002-2018-ds0001
