# Supplementary material for: Measuring rurality in health services research: a scoping review
Source: BMC Health Serv Res. 2022 Nov 12;22:1340. doi: 10.1186/s12913-022-08678-9 (PMC9652888; doi:10.1186/s12913-022-08678-9)
Supplement: Supplementary file 1 — Additional file 1. Appendix of included studies. [file 12913_2022_8678_MOESM1_ESM.docx]

| **Citation**  Appendix of included studies | **Geographic Unit** | **HSR Topic** | **Method/Definition** |
| --- | --- | --- | --- |
| Allen H, Wright B, Broffman L. The Impacts of Medicaid Expansion on Rural Low-Income Adults: Lessons From the Oregon Health Insurance Experiment. Med Care Res Rev. 2018 Jun;75(3):354-383. doi: 10.1177/1077558716688793. Epub 2017 Feb 3. PMID: 29148324; PMCID: PMC5726939. | County | Access | Rural Urban Continuum Codes |
| Anderson D, Abraham JM, Drake C. Rural-Urban Differences In Individual-Market Health Plan Affordability After Subsidy Payment Cuts. Health Aff (Millwood). 2019 Dec;38(12):2032-2040. doi: 10.1377/hlthaff.2019.00917. PMID: 31794305. | County | Cost | Other |
| Andrilla CHA, Patterson DG, Moore TE, Coulthard C, Larson EH. Projected Contributions of Nurse Practitioners and Physicians Assistant to Buprenorphine Treatment Services for Opioid Use Disorder in Rural Areas. Med Care Res Rev. 2020 Apr;77(2):208-216. doi: 10.1177/1077558718793070. Epub 2018 Aug 9. PMID: 30089426. | County | Access | Urban Influence Codes |
| Bailey BA, Manning T, Peiris AN. The impact of living in rural and urban areas: vitamin D and medical costs in veterans. J Rural Health. 2012 Fall;28(4):356-63. doi: 10.1111/j.1748-0361.2012.00407.x. PMID: 23017006. | County | Cost | Rural Urban Continuum Codes |
| Baldwin LM, Chan L, Andrilla CH, Huff ED, Hart LG. Quality of care for myocardial infarction in rural and urban hospitals. J Rural Health. 2010 Winter;26(1):51-7. doi: 10.1111/j.1748-0361.2009.00265.x. PMID: 20105268; PMCID: PMC3425799. | Zip Code | Quality | Rural Urban Commuting Area Codes |
| Barker AR. Effect Of Population Size On Rural Health Insurance Premiums In The Federal Employees Health Benefits Program. Health Aff (Millwood). 2019 Dec;38(12):2041-2047. doi: 10.1377/hlthaff.2019.00912. PMID: 31794303. | County | Cost | Urban Influence Codes |
| Barnes H, Richards MR, McHugh MD, Martsolf G. Rural And Nonrural Primary Care Physician Practices Increasingly Rely On Nurse Practitioners. Health Aff (Millwood). 2018 Jun;37(6):908-914. doi: 10.1377/hlthaff.2017.1158. PMID: 29863933; PMCID: PMC6080248. | County | Access | Core Based Statistical Areas |
| Barnett ML, Lee D, Frank RG. In Rural Areas, Buprenorphine Waiver Adoption Since 2017 Driven By Nurse Practitioners And Physician Assistants. Health Aff (Millwood). 2019 Dec;38(12):2048-2056. doi: 10.1377/hlthaff.2019.00859. PMID: 31794302; PMCID: PMC6938159. | County | Access | National Center for Health Statistics |
| Beatty K, Heffernan M, Hale N, Meit M. Funding and Service Delivery in Rural and Urban Local US Health Departments in 2010 and 2016. Am J Public Health. 2020 Sep;110(9):1293-1299. doi: 10.2105/AJPH.2020.305757. Epub 2020 Jul 16. PMID: 32673110; PMCID: PMC7427251. | Zip Code | Quality | Rural Urban Commuting Area Codes |
| Borders TF, Booth BM, Stewart KE, Cheney AM, Curran GM. Rural/urban residence, access, and perceived need for treatment among African American cocaine users. J Rural Health. 2015 Winter;31(1):98-107. doi: 10.1111/jrh.12092. Epub 2014 Sep 11. PMID: 25213603; PMCID: PMC4280311. | County | Access | County Designation (Federal) |
| Bowblis JR, Meng H, Hyer K. The urban-rural disparity in nursing home quality indicators: the case of facility-acquired contractures. Health Serv Res. 2013 Feb;48(1):47-69. doi: 10.1111/j.1475-6773.2012.01431.x. Epub 2012 Jun 7. PMID: 22670847; PMCID: PMC3589954. | Zip Code | Quality | Rural Urban Commuting Area Codes |
| Brooks E, Dailey N, Bair B, Shore J. Rural women veterans demographic report: defining VA users' health and health care access in rural areas. J Rural Health. 2014 Spring;30(2):146-52. doi: 10.1111/jrh.12037. Epub 2013 Jul 19. PMID: 24689540. | Zip Code | Access | VA System |
| Brown JD, Goodin AJ, Talbert JC. Rural and Appalachian Disparities in Neonatal Abstinence Syndrome Incidence and Access to Opioid Abuse Treatment. J Rural Health. 2018 Dec;34(1):6-13. doi: 10.1111/jrh.12251. Epub 2017 Jul 7. PMID: 28685864; PMCID: PMC5752583. | County | Access | Core Based Statistical Areas |
| Bukowski LA, Blosnich J, Shipherd JC, Kauth MR, Brown GR, Gordon AJ. Exploring Rural Disparities in Medical Diagnoses Among Veterans With Transgender-related Diagnoses Utilizing Veterans Health Administration Care. Med Care. 2017 Sep;55 Suppl 9 Suppl 2:S97-S103. doi: 10.1097/MLR.0000000000000745. PMID: 28806372. | Zip Code | Quality | Rural Urban Commuting Area Codes |
| Burns ME, Dague L, DeLeire T, Dorsch M, Friedsam D, Leininger LJ, Palmucci G, Schmelzer J, Voskuil K. The effects of expanding public insurance to rural low-income childless adults. Health Serv Res. 2014 Dec;49 Suppl 2(Suppl 2):2173-87. doi: 10.1111/1475-6773.12233. Epub 2014 Sep 27. PMID: 25262774; PMCID: PMC4241153. | County | Access | Metropolitan Statistical Areas |
| Butterfield PG, Hill W, Postma J, Butterfield PW, Odom-Maryon T. Effectiveness of a household environmental health intervention delivered by rural public health nurses. Am J Public Health. 2011 Dec;101 Suppl 1(Suppl 1):S262-70. doi: 10.2105/AJPH.2011.300164. Epub 2011 Aug 11. PMID: 21836117; PMCID: PMC3222509. | Zip Code | Access | Metropolitan Statistical Areas |
| Caldwell JT, Ford CL, Wallace SP, Wang MC, Takahashi LM. Intersection of Living in a Rural Versus Urban Area and Race/Ethnicity in Explaining Access to Health Care in the United States. Am J Public Health. 2016 Aug;106(8):1463-9. doi: 10.2105/AJPH.2016.303212. Epub 2016 Jun 16. PMID: 27310341; PMCID: PMC4940644. | Census Tract | Access | Rural Urban Commuting Area Codes |
| Casey MM, Hung P, Moscovice I, Prasad S. The Use of Hospitalists by Small Rural Hospitals: Results of a National Survey. Med Care Res Rev. 2014 Aug;71(4):356-66. doi: 10.1177/1077558714533822. Epub 2014 May 14. PMID: 24830380. | County | Other | Metropolitan Statistical Areas |
| Chandak A, Nayar P, Lin G. Rural-Urban Disparities in Access to Breast Cancer Screening: A Spatial Clustering Analysis. J Rural Health. 2019 Mar;35(2):229-235. doi: 10.1111/jrh.12308. Epub 2018 Jun 10. PMID: 29888497. | Census Tract | Access | Rural Urban Commuting Area Codes |
| Chen C, Xierali I, Piwnica-Worms K, Phillips R. The redistribution of graduate medical education positions in 2005 failed to boost primary care or rural training. Health Aff (Millwood). 2013 Jan;32(1):102-10. doi: 10.1377/hlthaff.2012.0032. PMID: 23297277. | County | Access | Rural Urban Continuum Codes |
| Chen HF, Landes RD, Schuldt RF, Tilford JM. Quality Performance of Rural and Urban Home Health Agencies: Implications for Rural Add-On Payment Policies. J Rural Health. 2020 Jun;36(3):423-432. doi: 10.1111/jrh.12415. Epub 2020 Feb 5. PMID: 32022948. | County | Quality | Other |
| Chen X, Orom H, Hay JL, Waters EA, Schofield E, Li Y, Kiviniemi MT. Differences in Rural and Urban Health Information Access and Use. J Rural Health. 2019 Jun;35(3):405-417. doi: 10.1111/jrh.12335. Epub 2018 Nov 16. PMID: 30444935; PMCID: PMC6522336. | County | Access | Core Based Statistical Areas |
| Cohen AJ, Ndoye M, Fergus KB, Lindsey J 2nd, Butler C, Patino G, Anger JT, Breyer BN. Forecasting Limited Access to Urology in Rural Communities: Analysis of the American Urological Association Census. J Rural Health. 2020 Jun;36(3):300-306. doi: 10.1111/jrh.12376. Epub 2019 May 24. PMID: 31125999. | Zip Code | Access | Rural Urban Commuting Area Codes |
| Cordasco KM, Mengeling MA, Yano EM, Washington DL. Health and Health Care Access of Rural Women Veterans: Findings From the National Survey of Women Veterans. J Rural Health. 2016 Sep;32(4):397-406. doi: 10.1111/jrh.12197. Epub 2016 Jul 28. PMID: 27466970. | County | Access | Core Based Statistical Areas |
| Crouch E, Eberth JM, Probst JC, Bennett K, Adams SA. Rural-Urban Differences in Costs of End-of-Life Care for the Last 6 Months of Life Among Patients with Breast, Lung, or Colorectal Cancer. J Rural Health. 2019 Mar;35(2):199-207. doi: 10.1111/jrh.12301. Epub 2018 Apr 15. PMID: 29656565. | County | Cost | Urban Influence Codes |
| Davis MM, Spurlock M, Dulacki K, Meath T, Li HF, McCarty D, Warne D, Wright B, McConnell KJ. Disparities in Alcohol, Drug Use, and Mental Health Condition Prevalence and Access to Care in Rural, Isolated, and Reservation Areas: Findings From the South Dakota Health Survey. J Rural Health. 2016 Jun;32(3):287-302. doi: 10.1111/jrh.12157. Epub 2015 Oct 30. PMID: 26515583; PMCID: PMC7331464. | Zip Code | Access | Rural Urban Commuting Area Codes |
| Del Rosario C, Kutney-Lee A, Sochalski J, Ersek M. Does Quality of End-of-Life Care Differ by Urban-Rural Location? A Comparison of Processes and Family Evaluations of Care in the VA. J Rural Health. 2019 Sep;35(4):528-539. doi: 10.1111/jrh.12351. Epub 2019 Feb 11. PMID: 30742330; PMCID: PMC6689442. | Zip Code | Quality | Rural Urban Commuting Area Codes |
| Doescher MP, Andrilla CH, Skillman SM, Morgan P, Kaplan L. The contribution of physicians, physician assistants, and nurse practitioners toward rural primary care: findings from a 13-state survey. Med Care. 2014 Jun;52(6):549-56. doi: 10.1097/MLR.0000000000000135. PMID: 24824539. | Zip Code | Access | Rural Urban Commuting Area Codes |
| Done N, Herring B, Xu T. The effects of global budget payments on hospital utilization in rural Maryland. Health Serv Res. 2019 Jun;54(3):526-536. doi: 10.1111/1475-6773.13162. PMID: 31066468; PMCID: PMC6505416. | Zip Code | Access | Other |
| Earle-Richardson G, Scribani M, Scott E, May J, Jenkins P. A comparison of health, health behavior, and access between farm and nonfarm populations in rural New York state. J Rural Health. 2015 Spring;31(2):157-64. doi: 10.1111/jrh.12098. Epub 2014 Nov 14. PMID: 25399689. | County | Access | County Designation (Federal) |
| Faul M, Dailey MW, Sugerman DE, Sasser SM, Levy B, Paulozzi LJ. Disparity in naloxone administration by emergency medical service providers and the burden of drug overdose in US rural communities. Am J Public Health. 2015 Jul;105 Suppl 3(Suppl 3):e26-32. doi: 10.2105/AJPH.2014.302520. Epub 2015 Apr 23. PMID: 25905856; PMCID: PMC4455515. | Population Density | Access | Metropolitan Statistical Areas |
| Fortney JC, Harman JS, Xu S, Dong F. The association between rural residence and the use, type, and quality of depression care. J Rural Health. 2010 Summer;26(3):205-13. doi: 10.1111/j.1748-0361.2010.00290.x. PMID: 20633088. | County | Access | Metropolitan Statistical Areas |
| Geissler KH. Differences in referral patterns for rural primary care physicians from 2005 to 2016. Health Serv Res. 2020 Feb;55(1):94-102. doi: 10.1111/1475-6773.13244. Epub 2019 Dec 17. PMID: 31845328; PMCID: PMC6980956. | County | Access | National Center for Health Statistics |
| Germack HD, Kandrack R, Martsolf GR. When Rural Hospitals Close, The Physician Workforce Goes. Health Aff (Millwood). 2019 Dec;38(12):2086-2094. doi: 10.1377/hlthaff.2019.00916. PMID: 31794309. | County | Access | Rural Urban Continuum Codes |
| Gong G, Phillips SG, Hudson C, Curti D, Philips BU. Higher US Rural Mortality Rates Linked To Socioeconomic Status, Physician Shortages, And Lack Of Health Insurance. Health Aff (Millwood). 2019 Dec;38(12):2003-2010. doi: 10.1377/hlthaff.2019.00722. PMID: 31794316. | County | Access | National Center for Health Statistics |
| Graves JM, Mackelprang JL, Moore M, Abshire DA, Rivara FP, Jimenez N, Fuentes M, Vavilala MS. Rural-urban disparities in health care costs and health service utilization following pediatric mild traumatic brain injury. Health Serv Res. 2019 Apr;54(2):337-345. doi: 10.1111/1475-6773.13096. Epub 2018 Dec 3. PMID: 30507042; PMCID: PMC6407359. | County | Cost | Metropolitan Statistical Areas |
| Greenberg AJ, Haney D, Blake KD, Moser RP, Hesse BW. Differences in Access to and Use of Electronic Personal Health Information Between Rural and Urban Residents in the United States. J Rural Health. 2018 Feb;34 Suppl 1(Suppl 1):s30-s38. doi: 10.1111/jrh.12228. Epub 2017 Jan 11. PMID: 28075508; PMCID: PMC5505819. | County | Access | Rural Urban Continuum Codes |
| Greenwood-Ericksen MB, Rothenberg C, Mohr N, Andrea SD, Slesinger T, Osborn T, Whittle J, Goyal P, Tarrant N, Schuur JD, Yealy DM, Venkatesh A. Urban and Rural Emergency Department Performance on National Quality Metrics for Sepsis Care in the United States. J Rural Health. 2019 Sep;35(4):490-497. doi: 10.1111/jrh.12339. Epub 2018 Nov 28. PMID: 30488590. | Zip Code | Quality | Rural Urban Commuting Area Codes |
| Hale NL, Smith M, Hardin J, Brock-Martin A. Rural populations and early periodic screening, diagnosis, and treatment services: challenges and opportunities for local public health departments. Am J Public Health. 2015 Apr;105 Suppl 2(Suppl 2):S330-6. doi: 10.2105/AJPH.2014.302449. Epub 2015 Feb 17. PMID: 25689205; PMCID: PMC4355708. | County | Access | Urban Influence Codes |
| Han X, Ku L. Enhancing Staffing In Rural Community Health Centers Can Help Improve Behavioral Health Care. Health Aff (Millwood). 2019 Dec;38(12):2061-2068. doi: 10.1377/hlthaff.2019.00823. PMID: 31794314. | Other | Access | Rural Urban Commuting Area Codes |
| Hearld LR, Carroll NW. Interorganizational Relationship Trends of Critical Access Hospitals. J Rural Health. 2016 Winter;32(1):44-55. doi: 10.1111/jrh.12131. Epub 2015 Jul 15. PMID: 26184387. | County | Other | Rural Urban Continuum Codes |
| Heisey-Grove DM. Variation In Rural Health Information Technology Adoption And Use. Health Aff (Millwood). 2016 Feb;35(2):365-70. doi: 10.1377/hlthaff.2015.0861. Epub 2016 Jan 20. PMID: 26791835. | County | Access | National Center for Health Statistics |
| Henning-Smith C, Kozhimannil K, Casey M, Prasad S, Moscovice I. Rural-Urban Differences in Medicare Quality Outcomes and the Impact of Risk Adjustment. Med Care. 2017 Sep;55(9):823-829. doi: 10.1097/MLR.0000000000000761. PMID: 28800000. | County | Quality | County Designation (Federal) |
| Henning-Smith C, Prasad S, Casey M, Kozhimannil K, Moscovice I. Rural-Urban Differences in Medicare Quality Scores Persist After Adjusting for Sociodemographic and Environmental Characteristics. J Rural Health. 2019 Jan;35(1):58-67. doi: 10.1111/jrh.12261. Epub 2017 Sep 5. PMID: 30561839. | County | Quality | National Center for Health Statistics |
| Holub JL, Morris C, Fagnan LJ, Logan JR, Michaels LC, Lieberman DA. Quality of Colonoscopy Performed in Rural Practice: Experience From the Clinical Outcomes Research Initiative and the Oregon Rural Practice-Based Research Network. J Rural Health. 2018 Feb;34 Suppl 1(Suppl 1):s75-s83. doi: 10.1111/jrh.12231. Epub 2017 Jan 3. PMID: 28045200; PMCID: PMC5495627. | Population Density | Quality | Rural Urban Commuting Area Codes |
| Horwitz JR, Nichols A. Rural hospital ownership: medical service provision, market mix, and spillover effects. Health Serv Res. 2011 Oct;46(5):1452-72. doi: 10.1111/j.1475-6773.2011.01280.x. Epub 2011 Jun 3. PMID: 21639860; PMCID: PMC3207187. | Zip Code | Cost | Metropolitan Statistical Areas |
| Hung P, Henning-Smith CE, Casey MM, Kozhimannil KB. Access To Obstetric Services In Rural Counties Still Declining, With 9 Percent Losing Services, 2004-14. Health Aff (Millwood). 2017 Sep 1;36(9):1663-1671. doi: 10.1377/hlthaff.2017.0338. Erratum in: Health Aff (Millwood). 2018 Apr;37(4):679. PMID: 28874496. | County | Access | County Designation (Federal) |
| Johnson LA, Melendez C, Larson K, Moye J, Schreier AM, Ellis C. Using Demographics to Predict Palliative Care Access in Inpatient Facilities in Rural North Carolina. J Rural Health. 2021 Mar;37(2):412-416. doi: 10.1111/jrh.12507. Epub 2020 Aug 18. PMID: 32808716. | Zip Code | Access | National Center for Health Statistics |
| Johnston KJ, Wen H, Joynt Maddox KE. Lack Of Access To Specialists Associated With Mortality And Preventable Hospitalizations Of Rural Medicare Beneficiaries. Health Aff (Millwood). 2019 Dec;38(12):1993-2002. doi: 10.1377/hlthaff.2019.00838. PMID: 31794307. | County | Access | Core Based Statistical Areas |
| Kaufman BG, Reiter KL, Pink GH, Holmes GM. Medicaid Expansion Affects Rural And Urban Hospitals Differently. Health Aff (Millwood). 2016 Sep 1;35(9):1665-72. doi: 10.1377/hlthaff.2016.0357. PMID: 27605649. | Zip Code | Cost | Rural Urban Commuting Area Codes |
| Kaufman BG, Reiter KL, Pink GH, Holmes GM. Medicaid Expansion Affects Rural And Urban Hospitals Differently. Health Aff (Millwood). 2016 Sep 1;35(9):1665-72. doi: 10.1377/hlthaff.2016.0357. PMID: 27605649. | County | Cost | County Designation (Federal) |
| Khan A, Thapa JR, Zhang D. Preventive Dental Checkups and Their Association With Access to Usual Source of Care Among Rural and Urban Adult Residents. J Rural Health. 2017 Sep;33(4):419-426. doi: 10.1111/jrh.12271. Epub 2017 Sep 14. PMID: 28905468. | County | Access | National Center for Health Statistics |
| Kilany M, Morrissey JP, Domino ME, Thomas KC, Silberman P. Utilization and Adherence in Medical Homes: An Assessment of Rural-Urban Differences for People With Severe Mental Illness. Med Care. 2018 Oct;56(10):870-876. doi: 10.1097/MLR.0000000000000973. PMID: 30211809; PMCID: PMC6298219. | County | Access | Rural Urban Continuum Codes |
| Kilmer G, Bynum L, Balamurugan A. Access to and use of eye care services in rural arkansas. J Rural Health. 2010 Winter;26(1):30-5. doi: 10.1111/j.1748-0361.2009.00262.x. PMID: 20105265. | County | Access | Metropolitan Statistical Areas |
| Kirby JB, Zuvekas SH, Borsky AE, Ngo-Metzger Q. Rural Residents With Mental Health Needs Have Fewer Care Visits Than Urban Counterparts. Health Aff (Millwood). 2019 Dec;38(12):2057-2060. doi: 10.1377/hlthaff.2019.00369. PMID: 31794321. | County | Access | Metropolitan Statistical Areas |
| Kirchhoff AC, Hart G, Campbell EG. Rural and urban primary care physician professional beliefs and quality improvement behaviors. J Rural Health. 2014 Summer;30(3):235-43. doi: 10.1111/jrh.12067. Epub 2014 Feb 16. PMID: 24528129. | Zip Code | Quality | Rural Urban Commuting Area Codes |
| Kozhimannil KB, Hung P, Prasad S, Casey M, McClellan M, Moscovice IS. Birth volume and the quality of obstetric care in rural hospitals. J Rural Health. 2014 Fall;30(4):335-43. doi: 10.1111/jrh.12061. Epub 2014 Feb 1. PMID: 24483138. | County | Quality | County Designation (Federal) |
| Kozhimannil KB, Hung P, Prasad S, Casey M, Moscovice I. Rural-urban differences in obstetric care, 2002-2010, and implications for the future. Med Care. 2014 Jan;52(1):4-9. doi: 10.1097/MLR.0000000000000016. PMID: 24316869; PMCID: PMC4130377. | County | Quality | Core Based Statistical Areas |
| Kozhimannil KB, Interrante JD, Henning-Smith C, Admon LK. Rural-Urban Differences In Severe Maternal Morbidity And Mortality In The US, 2007-15. Health Aff (Millwood). 2019 Dec;38(12):2077-2085. doi: 10.1377/hlthaff.2019.00805. PMID: 31794322. | Zip Code | Access | Metropolitan Statistical Areas |
| Kozhimannil KB, Interrante JD, Tuttle MS, Henning-Smith C, Admon L. Characteristics of US Rural Hospitals by Obstetric Service Availability, 2017. Am J Public Health. 2020 Sep;110(9):1315-1317. doi: 10.2105/AJPH.2020.305695. Epub 2020 Jul 16. PMID: 32673119; PMCID: PMC7427259. | County | Access | Metropolitan Statistical Areas |
| Krakow M, Hesse BW, Oh A, Patel V, Vanderpool RC, Jacobsen PB. Addressing Rural Geographic Disparities Through Health IT: Initial Findings From the Health Information National Trends Survey. Med Care. 2019 Jun;57 Suppl 6 Suppl 2:S127-S132. doi: 10.1097/MLR.0000000000001028. PMID: 31095051. | County | Access | Rural Urban Continuum Codes |
| Krishna S, Gillespie KN, McBride TM. Diabetes burden and access to preventive care in the rural United States. J Rural Health. 2010 Winter;26(1):3-11. doi: 10.1111/j.1748-0361.2009.00259.x. PMID: 20105262. | County | Access | Metropolitan Statistical Areas |
| Loftus J, Allen EM, Call KT, Everson-Rose SA. Rural-Urban Differences in Access to Preventive Health Care Among Publicly Insured Minnesotans. J Rural Health. 2018 Feb;34 Suppl 1(Suppl 1):s48-s55. doi: 10.1111/jrh.12235. Epub 2017 Mar 14. PMID: 28295584; PMCID: PMC6069955. | County | Access | Metropolitan Statistical Areas |
| Lund BC, Charlton ME, Steinman MA, Kaboli PJ. Regional differences in prescribing quality among elder veterans and the impact of rural residence. J Rural Health. 2013 Spring;29(2):172-9. doi: 10.1111/j.1748-0361.2012.00428.x. Epub 2012 Aug 27. PMID: 23551647; PMCID: PMC3940354. | Census Tract | Quality | VA Sytem |
| Martsolf GR, Baird M, Cohen CC, Koirala N. Relationship Between State Policy and Anesthesia Provider Supply in Rural Communities. Med Care. 2019 May;57(5):341-347. doi: 10.1097/MLR.0000000000001106. PMID: 30870391. | County | Access | Rural Urban Continuum Codes |
| McCarthy JF, Blow FC, Ignacio RV, Ilgen MA, Austin KL, Valenstein M. Suicide among patients in the Veterans Affairs health system: rural-urban differences in rates, risks, and methods. Am J Public Health. 2012 Mar;102 Suppl 1(Suppl 1):S111-7. doi: 10.2105/AJPH.2011.300463. Epub 2012 Jan 25. PMID: 22390583; PMCID: PMC3496440. | Zip Code | Access | VA System |
| McFarland KK, Salama F, Yaseen M. Access to dental care for rural children: a survey of Nebraska general dentists. J Rural Health. 2011 Spring;27(2):205-10. doi: 10.1111/j.1748-0361.2010.00333.x. Epub 2010 Sep 27. PMID: 21457314. | Population Density | Access | County Designation (State) |
| Miller KEM, James HJ, Holmes GM, Van Houtven CH. The effect of rural hospital closures on emergency medical service response and transport times. Health Serv Res. 2020 Apr;55(2):288-300. doi: 10.1111/1475-6773.13254. Epub 2020 Jan 27. PMID: 31989591; PMCID: PMC7080401. | Zip Code | Quality | Urban Influence Codes |
| Mitchell J, Probst J, Brock-Martin A, Bennett K, Glover S, Hardin J. Association between clinical decision support system use and rural quality disparities in the treatment of pneumonia. J Rural Health. 2014 Spring;30(2):186-95. doi: 10.1111/jrh.12043. Epub 2013 Aug 12. PMID: 24689543. | Zip Code | Quality | Other |
| Morden NE, Berke EM, Welsh DE, McCarthy JF, Mackenzie TA, Kilbourne AM. Quality of care for cardiometabolic disease: associations with mental disorder and rurality. Med Care. 2010 Jan;48(1):72-8. doi: 10.1097/MLR.0b013e3181bd49f7. PMID: 19927015; PMCID: PMC4057647. | Zip Code | Other | Rural Urban Commuting Area Codes |
| Mroz TM, Patterson DG, Frogner BK. The Impact Of Medicare's Rural Add-On Payments On Supply Of Home Health Agencies Serving Rural Counties. Health Aff (Millwood). 2020 Jun;39(6):949-957. doi: 10.1377/hlthaff.2019.00952. PMID: 32479227; PMCID: PMC8510476. | County | Cost | Rural Urban Commuting Area Codes |
| Neprash HT, Smith LB, Sheridan B, Moscovice I, Prasad S, Kozhimannil K. Nurse Practitioner Autonomy and Complexity of Care in Rural Primary Care. Med Care Res Rev. 2021 Dec;78(6):684-692. doi: 10.1177/1077558720945913. Epub 2020 Jul 29. PMID: 32727272. | County | Other | County Designation (Federal) |
| Newhouse RP, Dennison Himmelfarb C, Morlock L, Frick KD, Pronovost P, Liang Y. A phased cluster-randomized trial of rural hospitals testing a quality collaborative to improve heart failure care: organizational context matters. Med Care. 2013 May;51(5):396-403. doi: 10.1097/MLR.0b013e318286e32e. PMID: 23579349. | Other | Quality | Other |
| Njei B, Esserman D, Krishnan S, Ohl M, Tate JP, Hauser RG, Taddei T, Lim J, Justice AC. Regional and Rural-Urban Differences in the Use of Direct-acting Antiviral Agents for Hepatitis C Virus: The Veteran Birth Cohort. Med Care. 2019 Apr;57(4):279-285. doi: 10.1097/MLR.0000000000001071. PMID: 30807449; PMCID: PMC6436819. | Zip Code | Access | Rural Urban Commuting Area Codes |
| Noles MJ, Reiter KL, Boortz-Marx J, Pink G. Rural Hospital Mergers and Acquisitions: Which Hospitals Are Being Acquired and How Are They Performing Afterward? J Healthc Manag. 2015 Nov-Dec;60(6):395-407. PMID: 26720983. | County | Other | County Designation (Federal) |
| O'Hanlon CE, Kranz AM, DeYoreo M, Mahmud A, Damberg CL, Timbie J. Access, Quality, And Financial Performance Of Rural Hospitals Following Health System Affiliation. Health Aff (Millwood). 2019 Dec;38(12):2095-2104. doi: 10.1377/hlthaff.2019.00918. PMID: 31794306; PMCID: PMC7004480. | County | Quality | County Designation (Federal) |
| Ohl M, Tate J, Duggal M, Skanderson M, Scotch M, Kaboli P, Vaughan-Sarrazin M, Justice A. Rural residence is associated with delayed care entry and increased mortality among veterans with human immunodeficiency virus infection. Med Care. 2010 Dec;48(12):1064-70. doi: 10.1097/MLR.0b013e3181ef60c2. PMID: 20966783; PMCID: PMC3138500. | Zip Code | Access | Rural Urban Commuting Area Codes |
| Ohl ME, Richardson K, Kaboli PJ, Perencevich EN, Vaughan-Sarrazin M. Geographic access and use of infectious diseases specialty and general primary care services by veterans with HIV infection: implications for telehealth and shared care programs. J Rural Health. 2014 Fall;30(4):412-21. doi: 10.1111/jrh.12070. Epub 2014 Apr 6. PMID: 24702698. | Zip Code | Access | Rural Urban Commuting Area Codes |
| Onega T, Duell EJ, Shi X, Demidenko E, Goodman D. Influence of place of residence in access to specialized cancer care for African Americans. J Rural Health. 2010 Winter;26(1):12-9. doi: 10.1111/j.1748-0361.2009.00260.x. PMID: 20105263. | Zip Code | Access | Rural Urban Commuting Area Codes |
| Owsley KM, Hamer MK, Mays GP. The Growing Divide in the Composition of Public Health Delivery Systems in US Rural and Urban Communities, 2014-2018. Am J Public Health. 2020 Jul;110(S2):S204-S210. doi: 10.2105/AJPH.2020.305801. PMID: 32663081; PMCID: PMC7362689. | County | Access | Rural Urban Commuting Area Codes |
| Patel SY, Huskamp HA, Busch AB, Mehrotra A. Telemental Health and US Rural-Urban Differences in Specialty Mental Health Use, 2010-2017. Am J Public Health. 2020 Sep;110(9):1308-1314. doi: 10.2105/AJPH.2020.305657. Epub 2020 Jul 16. PMID: 32673109; PMCID: PMC7427215. | Other | Access | Rural Urban Commuting Area Codes |
| Phipps MS, Jia H, Chumbler NR, Li X, Castro JG, Myers J, Williams LS, Bravata DM. Rural-urban differences in inpatient quality of care in US Veterans with ischemic stroke. J Rural Health. 2014 Winter;30(1):1-6. doi: 10.1111/jrh.12029. Epub 2013 Jun 6. PMID: 24383479. | Zip Code | Quality | Rural Urban Commuting Area Codes |
| Pilkey D, Edwards C, Richards R, Olson LM, Ely M, Edgerton EA. Pediatric Readiness in Critical Access Hospital Emergency Departments. J Rural Health. 2019 Sep;35(4):480-489. doi: 10.1111/jrh.12317. Epub 2018 Jul 30. PMID: 30062684. | Zip Code | Access | Other |
| Probst JC, Bellinger JD, Walsemann KM, Hardin J, Glover SH. Higher risk of death in rural blacks and whites than urbanites is related to lower incomes, education, and health coverage. Health Aff (Millwood). 2011 Oct;30(10):1872-9. doi: 10.1377/hlthaff.2011.0668. PMID: 21976329. | County | Access | Urban Influence Codes |
| Reiter KL, Noles M, Pink GH. Uncompensated Care Burden May Mean Financial Vulnerability For Rural Hospitals In States That Did Not Expand Medicaid. Health Aff (Millwood). 2015 Oct;34(10):1721-9. doi: 10.1377/hlthaff.2014.1340. PMID: 26438749. | County | Cost | Metropolitan Statistical Areas |
| Richards MR, Saloner B, Kenney GM, Rhodes KV, Polsky D. Availability of New Medicaid Patient Appointments and the Role of Rural Health Clinics. Health Serv Res. 2016 Apr;51(2):570-91. doi: 10.1111/1475-6773.12334. Epub 2015 Jun 26. PMID: 26119695; PMCID: PMC4799893. | County | Access | National Center for Health Statistics |
| Rodriguez Villalvazo Y, McDanel JS, Beste LA, Sanchez AJ, Vaughan-Sarrazin M, Katz DA. Effect of travel distance and rurality of residence on initial surveillance for hepatocellular carcinoma in VA primary care patient with cirrhosis. Health Serv Res. 2020 Feb;55(1):103-112. doi: 10.1111/1475-6773.13241. Epub 2019 Nov 25. PMID: 31763691; PMCID: PMC6980959. | Zip Code | Access | Rural Urban Commuting Area Codes |
| Spetz J, Skillman SM, Andrilla CHA. Nurse Practitioner Autonomy and Satisfaction in Rural Settings. Med Care Res Rev. 2017 Apr;74(2):227-235. doi: 10.1177/1077558716629584. Epub 2016 Aug 3. PMID: 26825943. | Zip Code | Other | Rural Urban Commuting Area Codes |
| Stensland J, Akamigbo A, Glass D, Zabinski D. Rural and urban Medicare beneficiaries use remarkably similar amounts of health care services. Health Aff (Millwood). 2013 Nov;32(11):2040-6. doi: 10.1377/hlthaff.2013.0693. Epub 2013 Oct 30. PMID: 24173368. | County | Access | Urban Influence Codes |
| Thomas LV, Wedel KR, Christopher JE. Access to Transportation and Health Care Visits for Medicaid Enrollees With Diabetes. J Rural Health. 2018 Mar;34(2):162-172. doi: 10.1111/jrh.12239. Epub 2017 Mar 28. PMID: 28370462. | Zip Code | Access | Rural Urban Commuting Area Codes |
| Tonks SA, Makwana S, Salanitro AH, Safford MM, Houston TK, Allison JJ, Curry W, Estrada CA. Quality of diabetes mellitus care by rural primary care physicians. J Rural Health. 2012 Fall;28(4):364-71. doi: 10.1111/j.1748-0361.2012.00410.x. Epub 2012 May 31. PMID: 23083082; PMCID: PMC3481192. | Zip Code | Quality | Rural Urban Commuting Area Codes |
| Toth M, Holmes M, Toles M, Van Houtven C, Weinberger M, Silberman P. Impact of Postdischarge Follow-Up Care on Medicare Expenditures: Does Rural Make a Difference? Med Care Res Rev. 2018 Jun;75(3):327-353. doi: 10.1177/1077558716687499. Epub 2017 Jan 19. PMID: 29148323. | Other | Cost | Rural Urban Commuting Area Codes |
| Toth M, Holmes M, Van Houtven C, Toles M, Weinberger M, Silberman P. Rural-Urban Differences in the Effect of Follow-Up Care on Postdischarge Outcomes. Health Serv Res. 2017 Aug;52(4):1473-1493. doi: 10.1111/1475-6773.12543. Epub 2016 Aug 8. PMID: 27500788; PMCID: PMC5517676. | County | Cost | Urban Influence Codes |
| Toth M, Holmes M, Van Houtven C, Toles M, Weinberger M, Silberman P. Rural Medicare Beneficiaries Have Fewer Follow-up Visits and Greater Emergency Department Use Postdischarge. Med Care. 2015 Sep;53(9):800-8. doi: 10.1097/MLR.0000000000000401. Erratum in: Med Care. 2015 Oct;53(10):908. PMID: 26270827. | Other | Cost | Rural Urban Commuting Area Codes |
| Wang H, Qiu F, Boilesen E, Nayar P, Lander L, Watkins K, Watanabe-Galloway S. Rural-Urban Differences in Costs of End-of-Life Care for Elderly Cancer Patients in the United States. J Rural Health. 2016 Sep;32(4):353-362. doi: 10.1111/jrh.12160. Epub 2015 Nov 20. PMID: 26586101. | County | Cost | Urban Influence Codes |
| Watanabe-Galloway S, Zhang W, Watkins K, Islam KM, Nayar P, Boilesen E, Lander L, Wang H, Qiu F. Quality of end-of-life care among rural Medicare beneficiaries with colorectal cancer. J Rural Health. 2014 Fall;30(4):397-405. doi: 10.1111/jrh.12074. Epub 2014 May 6. PMID: 24803384. | County | Quality | Urban Influence Codes |
| Weigel PAM, Ullrich F, Ward MM. Rural Bypass of Critical Access Hospitals in Iowa: Do Visiting Surgical Specialists Make a Difference? J Rural Health. 2018 Feb;34 Suppl 1:s21-s29. doi: 10.1111/jrh.12220. Epub 2016 Sep 28. PMID: 27677870. | Zip Code | Access | Other |
| Whitacre BE. The Influence of the Degree of Rurality on EMR Adoption, by Physician Specialty. Health Serv Res. 2017 Apr;52(2):616-633. doi: 10.1111/1475-6773.12510. Epub 2016 Jun 3. PMID: 27256561; PMCID: PMC5346494. | County | Access | Rural Urban Continuum Codes |
| Williams D Jr, Pink GH, Song PH, Reiter KL, Holmes GM. Capital Expenditures Increased at Rural Hospitals That Merged Between 2012 and 2015. J Healthc Manag. 2020 Sep-Oct;65(5):346-364. doi: 10.1097/JHM-D-19-00219. PMID: 32925534. | County | Cost | County Designation (Federal) |
| Wright B, Jung HY, Feng Z, Mor V. Trends in observation care among Medicare fee-for-service beneficiaries at critical access hospitals, 2007-2009. J Rural Health. 2013 Aug;29 Suppl 1(0 1):s1-6. doi: 10.1111/jrh.12007. Epub 2013 Feb 22. PMID: 23944275; PMCID: PMC3752707. | County | Quality | National Center for Health Statistics |
| Zhang D, Wang G, Zhu W, Thapa JR, Switzer JA, Hess DC, Smith ML, Ritchey MD. Expansion Of Telestroke Services Improves Quality Of Care Provided In Super Rural Areas. Health Aff (Millwood). 2018 Dec;37(12):2005-2013. doi: 10.1377/hlthaff.2018.05089. PMID: 30633675. | Population Density | Access | Metropolitan Statistical Areas |
| Zhu X, Mueller K, Huang H, Ullrich F, Vaughn T, MacKinney AC. Organizational Attributes Associated With Medicare ACO Quality Performance. J Rural Health. 2019 Jan;35(1):68-77. doi: 10.1111/jrh.12304. Epub 2018 May 8. PMID: 29737573. | County | Quality | Urban Influence Codes |
| Ziller EC, Anderson NJ, Coburn AF. Access to rural mental health services: service use and out-of-pocket costs. J Rural Health. 2010 Summer;26(3):214-24. doi: 10.1111/j.1748-0361.2010.00291.x. PMID: 20633089. | County | Cost | Rural Urban Continuum Codes |
